# Impact of microbial genome completeness on metagenomic functional inference

**DOI:** 10.1038/s43705-023-00221-z

**Published:** 2023-02-16

**Authors:** Raphael Eisenhofer, Iñaki Odriozola, Antton Alberdi

**Affiliations:** Center for Evolutionary Hologenomics, Globe Institute, University of Copenhagen, Copenhagen, Denmark

**Keywords:** Bacterial genetics, Bacterial genetics

## Abstract

Inferring the functional capabilities of bacteria from metagenome-assembled genomes (MAGs) is becoming a central process in microbiology. Here we show that the completeness of genomes has a significant impact on the recovered functional signal, spanning all domains of metabolic functions. We identify factors that affect this relationship between genome completeness and function fullness, and provide baseline knowledge to guide efforts to correct for this overlooked bias in metagenomic functional inference.

## Background

Genome-resolved metagenomics enables draft bacterial genomes to be reconstructed from DNA sequence data derived from complex microbial mixtures [1]. The metagenome-assembled genomes (MAG) derived from such a process can be annotated to predict their functional toolbox upon which microbiome-level functional analyses can be conducted [2, 3]. While long-read sequencing technologies can recover circularised genomes from metagenomic mixtures [4], one of the main issues of short-read sequencing-based approaches is that MAGs usually display different levels of genome completeness, i.e., the entirety of a microbe’s DNA is not always captured in the reconstructed genome [5]. Genome completeness is primarily estimated through the presence of single-copy core genes (SCCGs), which are expected to be found in most bacteria [6]. It is common to use MAGs with completeness values as low as 70% for the functional analyses of microbial communities [7]. However, if a genome is estimated to be 70% complete, it is probable that many of the functions encoded in the actual genome will not be captured in the MAG, and thus the functional capacity of the genome will be underestimated [3, 8]. Not accounting for the level of completeness of MAGs could therefore lead researchers to incorrect interpretations of results, such as the artifactual deficit of functions being misinterpreted as real biological signal.

**Fig. 1:**
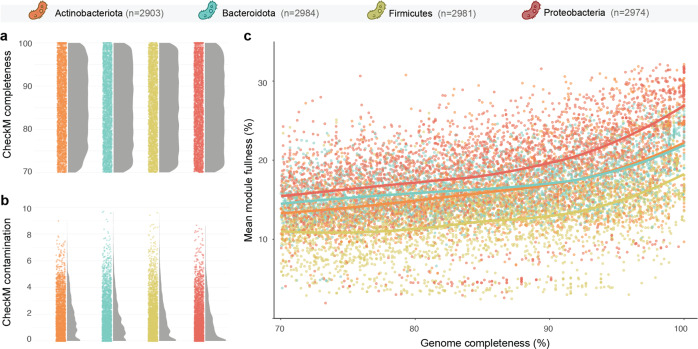
General genome completeness, contamination and metabolic module fullness statistics. Distribution of CheckM completeness (**a**) and contamination (**b**) values of the genomes used in this study displayed as jitter plots and associated density curves. **c** Relationship between genome completeness and mean KEGG module fullness. Dots indicate individual genomes coloured by taxonomic phylum. Solid lines indicate LOESS smoothed mean for each phylum.

A major challenge of metagenomic research is correcting or accounting for biases in statistical analyses and modelling. However, we currently ignore how the loss of functional capacities is correlated with genome completeness, and whether these relationships are constant or variable across microbial phylogeny and metabolic domains. To address these issues, we conducted two complementary analyses. First, we investigated the relationship between estimated genome completeness and metabolic function fullness (defined as the proportion of biochemical reactions enabled by the genes present in a genome to accomplish a metabolic function) using 11,842 genomes from diverse origins publicly accessible at the GTDB database [9]. Genome completeness was estimated using CheckM [6], while functional fullness of KEGG modules was estimated using DRAM [2, 10]. To ensure robust statistical modelling based on unbiased data, only MAGs belonging to the four most diverse bacterial phyla were considered; namely, *Actinobacteriota*, *Bacteroidota*, *Firmicutes* and *Proteobacteria* (around 3000 genomes each). The representation of genome completeness was evenly distributed across 70–100%, each window of 1% containing ca. 100 genomes from each phylum (Fig. 1a), and only genomes with contamination/redundancy values under 10% were considered (Fig. 1b). We also filtered the KEGG modules used for the modelling, by only considering the functions represented in at least 5% of the genomes (i.e., minimum representation of 592 data points). Finally, using a mock community of eight bacterial species, we compared 240 incomplete genomes (subsampled from MAGs) to their circularised genome counterparts, and applied a naive correction to their functional profiles using the previously trained models to showcase the possibility to improve functional inferences from incomplete bacterial genomes.

## Results and discussion

**Fig. 2:**
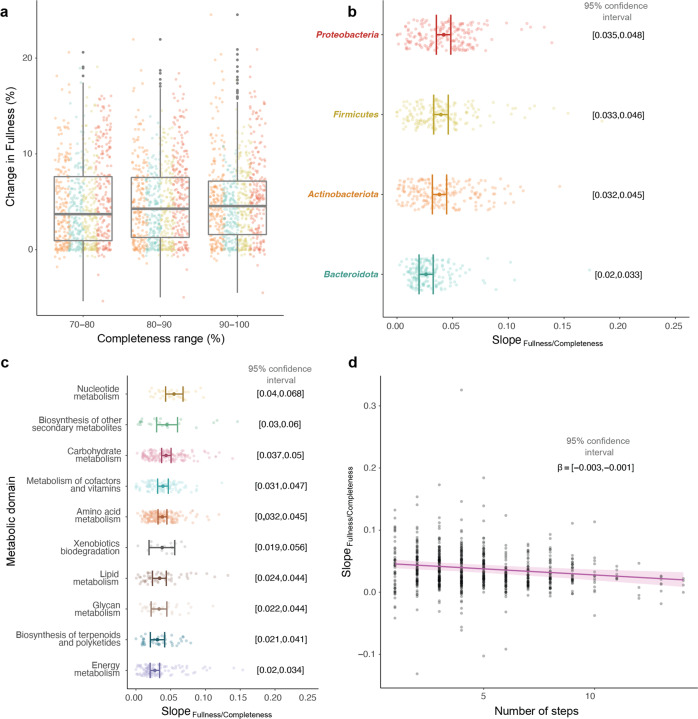
Relationship between function fullness and MAG completeness. **a** Percentage of change in functional module fullness across different genome completeness windows with dots coloured by phylum. **b** Mean slope variation of the fullness/completeness relationship across bacterial phyla. **c** Mean slope variation of the fullness/completeness relationship across functional domains. **d** Relationship between the slope of the fullness/completeness relationship and the number of steps of the modules.

We employed generalised linear models with binomial distribution to understand the association of genome completeness and function fullness in a filtered set of 195 KEGG metabolic modules across 11,842 genomes (Fig. 1c). The models estimated a positive relationship between genome completeness and metabolic function fullness for 94% of the studied modules, spanning all functional domains and levels of complexity (i.e., number of enzymatic steps). Overall, the increase of completeness from 70 to 100% was associated with a 15 ± 10% (mean ± sd) increase in module fullness. This relationship remained constant across the completeness gradient, with a slight tendency for the slope of the relationship to increase with completeness (Fig. 2a). This indicates that, while increasing the threshold to exclude MAGs with low completeness from functional analyses minimises the issue, the problem persists even when only considering ‘high quality’ (>90%) MAGs. We also found evidence for significant differences between the fullness-completeness relationship across bacterial phyla. Considering all KEGG modules analysed, *Proteobacteria* showed the overall strongest fullness-completeness relationship followed by *Firmicutes*, *Actinobacteriota* and *Bacteroidota* (Fig. 2b).

Similarly to taxonomic differences, the fullness-completeness relationship did not change evenly across metabolic domains. The fullness of the modules belonging to the ‘nucleotide metabolism’ and ‘biosynthesis of other secondary metabolites’ domains were the most affected by completeness, while ‘energy metabolism’ showed the weakest fullness-completeness association (Fig. 2c). In addition, the complexity of the modules was negatively associated with the fullness-completeness relationship (Fig. 2d). This suggests that the fullness of the modules with the fewest steps are the ones that are more severely affected by genome incompleteness.

**Fig. 3:**
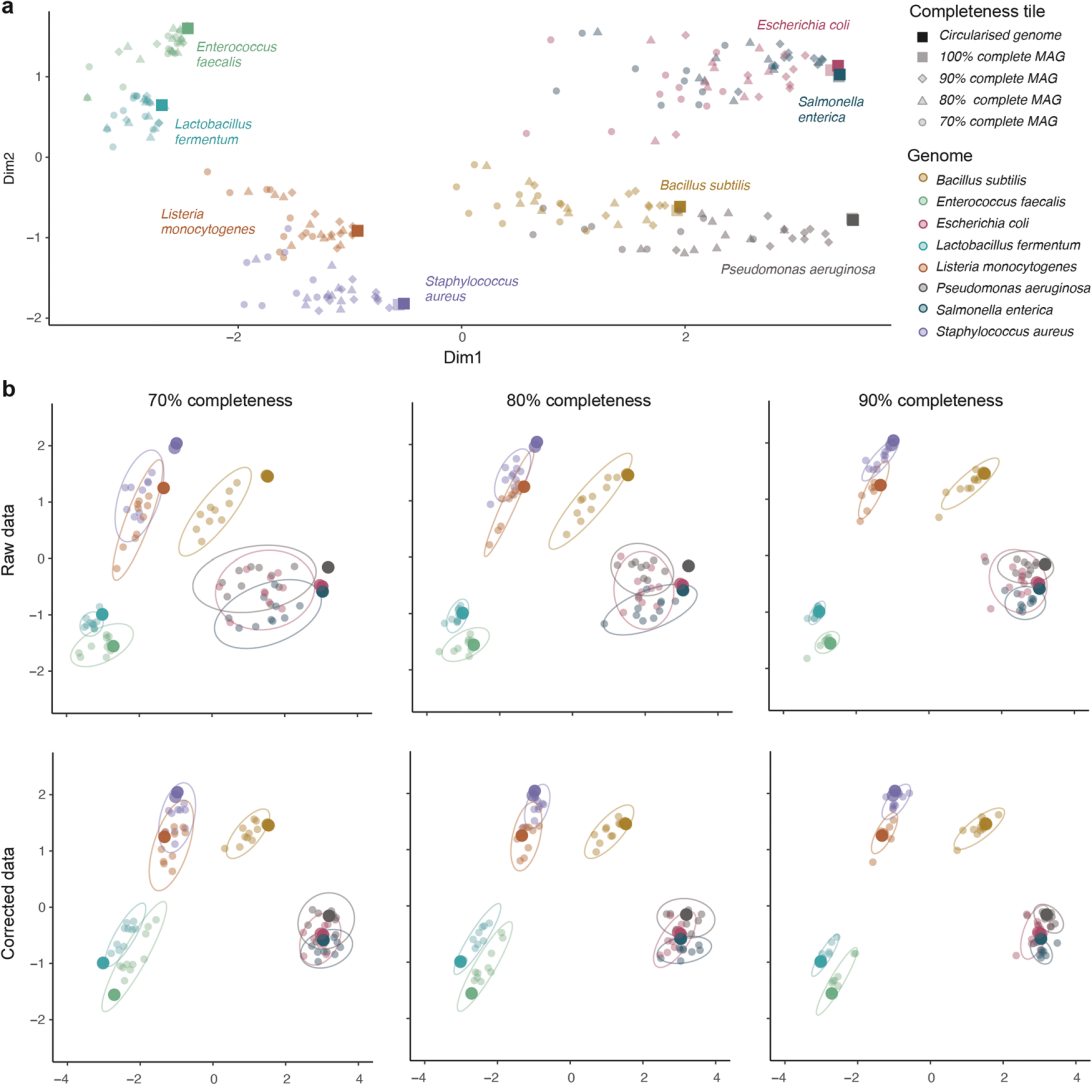
Principal coordinate analysis (PCoA) ordination of the functional profiles of eight complete genomes included in a mock community and their subsampled replicates. **a** Overall representation of all complete and subsampled genomes. Colours indicate bacterial genomes while shapes show their completeness level. Solid squares are complete circularised genomes while faded squares are their near-complete counterparts reconstructed from metagenomic data. Diamonds, triangles and circles indicate the completeness tile of subsampled MAGs. **b** Comparison between the raw and corrected functional profiles of genomes subsampled at 70%, 80 and 90% completeness. Ellipses show the 95% confidence interval of the ten replicates subsampled at each completeness tile. Solid circles refer to 100% genomes, which can be observed that fall more often within the 95% confidence interval ellipses of incomplete genomes when functional profiles are corrected, indicating corrected fullness values are closer to reality than the original ones.

We then implemented a complementary approach to assess the magnitude of the issue, by comparing the functional profiles of eight circularised genomes to their respective MAGs with ca. 70%, 80 and 90% completeness, as reconstructed and subsampled using genome-resolved metagenomics. The principal coordinate analysis (PCoA) of the genomes from the mock community revealed that reducing genome completeness from 100 to 70% introduced a systematic bias to their functional profiles of proportional magnitude to the level of the subsampling (Fig. 3a). A striking example is for the *Pseudomonas aeruginosa* genome, as genome incompleteness shifts its functional profile towards that of *Bacillus subtilis*.

Finally, as an initial attempt to showcase that completeness-biases can be minimised, we used the module-specific binomial generalised linear models trained using the MAGs from GTDB database to correct the functional profiles of incomplete MAGs. PCoA ordination showed that our models consistently reduced the functional bias introduced by genome incompleteness (Fig. 3b). The correction was rather successful for most genomes (the 95% confidence interval ellipses of the MAGs wrapped the complete genome), although it tended to ‘overcorrect’ others (e.g., *Enterococcus faecalis* and *Lactobacillus fermentum*).

## Conclusions

Our results highlight the need to consider genome completeness when comparing the functional capacities between microbial genomes or metagenomes. Although chromosomal information does not encode the entire functional toolbox of microorganisms [11], linking plasmids and other extrachromosomal elements to bacterial strains in metagenomic data faces other challenges that are beyond the scope of this article [12]. Currently, the focus of most genome-resolved metagenomic studies is limited to chromosomal DNA, and our results show that incorrect conclusions can be drawn if completeness biases are not considered. We argue that completeness biases should be accounted for in functional analyses based on metagenomics, analogously to how DNA sequencing depth biases are considered in diversity modelling approaches [13]. Although the aim of this study was to showcase the bias introduced by genome incompleteness rather than correcting it, our simple correction approach considerably reduced the bias and contributed to recover less distorted functional profiles of incomplete MAGs. However, we believe there is ample room for improvement by including more variables that contribute to explaining the link between genome completeness and function fullness. Only through the correction and mitigation of the functional biases introduced by uneven genome completeness will researchers be able to robustly characterise, model, and assess the functional capabilities of microbial communities.

## Materials and methods

### Genome retrieval, annotation and distillation

We browsed the GTDB database [9], which contains complete and draft bacterial genomes with associated CheckM completeness scores, for Bacterial phyla with at least 100 genomes in each of the 1% windows ranging 70–100% of genome completeness with <10% contamination. These criteria were met by four phyla, namely *Actinobacteriota*, *Bacteroidota*, *Firmicutes* (*sensu lato*, including *Bacilli* and *Clostridia*) and *Proteobacteria*, which were considered for analysis. Genomes within each completeness window and phylum were randomly selected, and their sequences retrieved from the NCBI database [14] using their assembly accession codes. For the subsampled complete genome analysis, a sequenced ZymoBIOMICS® Microbial Community Standard from Olm et al. [15] was downloaded using kingfisher download (https://github.com/wwood/kingfisher-download). This mock community contains 8 bacterial species that have complete reference genomes available. The downloaded reads were randomly subsampled to different depths (1 million, 2 million, 5 million, and ALL) using seqtk sample with a seed of 1337 (https://github.com/lh3/seqtk), and each processed through the ‘Individual_Assembly_Binning.snakefile’ pipeline (https://github.com/earthhologenome/EHI_bioinformatics). dRep [16] was then used on all resulting MAGs with the inclusion of the complete bacterial genome references to determine which MAG was from which bacterial genome. A representative MAG from each species (CheckM completeness >90, contamination <5, minimum at least 42 contigs) had contigs randomly subsampled ten times at three different rates (70, 80, 90% of contigs remaining) using BBMap’s reformat.sh (total of 240 MAG subsamplings). CheckM was then run on these subsampled MAGs to estimate completeness.

Genomes were subsequently annotated and distilled to KEGG pathway fullness values (defined as the proportion of biochemical reactions enabled by the genes present in a genome to accomplish a metabolic function) using DRAM (1.2.4) [2]. The summarise_genomes.py script was modified to output all modules and module numbers (https://github.com/EisenRa/DRAM_more_modules/tree/1.2.4_more_modules).

### Statistics and visualisation

Statistical analyses were conducted on KEGG module fullness data. Only widespread KEGG modules present in at least 5% of MAGs were used for statistical modelling. This filtering resulted in 195 KEGG modules observed across 11,842 MAGs. Statistical analyses were conducted in three consecutive steps.

In the first step, generalised linear models were used to estimate the relationship between fullness of KEGG modules and completeness of genomes. A binomial distribution was used with the logit link function, since function fullness represents the proportion of enzymatic reactions (or steps) of a module present in a genome. The total number of steps of each module were used as weights in the models. Genome completeness (numeric variable), the bacterial Phylum (categorical variable with four levels) and their interaction were used as fixed explanatory variables, thus a Phylum-specific slope was estimated for each module.

In the second step, linear mixed effect modelling, as implemented in the R package lme4 [17], was used to explore predictors of the strength of the fullness-completeness relationship across modules. The Phylum-specific slopes estimated in aforementioned binomial models were used as response variables and bacterial Phylum (categorical variable with four levels), the KEGG domain of each module (categorical variable with ten levels), and the number of steps involved in a module (numeric variable) were used as fixed explanatory variables. Since four slopes were estimated for each module (one for each bacterial Phylum) and they were included in the response variable for this model, a module-level random effect was included in the model as random intercept (random = 1|module). We built bootstrap confidence intervals around the levels of the categorical variables through the bootMER() function with 999 simulations, and around the slope of the numeric variable using the function confint.merMod(), both included in the lme4 package. To make the marginal predictions for each categorical variable, the non-focal categorical variables (Phylum or domain) were kept in their reference level and the numeric variable (steps) in its mean value. Non-overlapping 95% confidence intervals between the levels of the categorical factors Phylum and domain were considered as evidence against the null hypothesis of no differences between groups. Similarly, for the numeric variable number of steps, a confidence interval of the slope not overlapping zero was considered as evidence against the null hypothesis of no association.

In the third step, we first used the functional profiles of 8 genomes from a mock community and their subsampled replicates to perform a Principal Coordinate Analysis (PCoA) for visual assessment of the bias introduced by the reduction of genome completeness to around 90%, 80 and 70%. Then, we used the module-specific binomial generalised linear models constructed in step one (trained with the MAGs retrieved from GTDB database) to correct the functional profiles of the subsampled genomes. For this, we generated two predictions for each focal genome based on its Phylum: the predicted module fullness given its observed completeness, and the predicted module fullness if the genome was 100% complete. The difference between both predictions was added to the observed module fullness in each focal genome. If the corrected module fullness was larger than 1 it was rounded to 1. Lastly, a joint PCoA was conducted using the functional profiles of complete genomes, the raw subsampled genomes and the corrected subsampled genomes, and displayed in six plots for the sake of visualisation.

## Data Availability

Genome sequences and metadata employed in this study were retrieved from the NCBI and GTDB databases. A table containing the accession codes and metadata of the genomes, and the scripts employed for generating the results, are archived in Zenodo: https://zenodo.org/record/7584430, (10.5281/zenodo.7584429).
